# Towards Greener Polymers: Poly(octamethylene itaconate-*co*-succinate) Synthesis Parameters

**DOI:** 10.3390/polym17162220

**Published:** 2025-08-14

**Authors:** Magdalena Miętus, Tomasz Gołofit, Agnieszka Gadomska-Gajadhur

**Affiliations:** Faculty of Chemistry, Warsaw University of Technology, Noakowskiego 3 Street, 00-664 Warsaw, Poland; magdalena.mietus.dokt@pw.edu.pl (M.M.); tomasz.golofit@pw.edu.pl (T.G.)

**Keywords:** mathematical optimization, itaconic acid, design of experiments

## Abstract

A group of renewable, unsaturated resins from itaconic acid, 1,8-octanediol, and succinic anhydride were synthesized in a non-solvent and non-catalyst melt polycondensation reaction. The study addresses the need for sustainable polymeric materials suitable for additive manufacturing by investigating the influence of synthesis parameters—namely itaconic acid content, reaction time, and temperature—on the properties of poly(octamethylene itaconate-*co*-succinate) (POItcSc). The Box-Behnken mathematical planning method was applied to optimize the reaction conditions. The optimal synthesis conditions of POItcSc were achieved with an itaconic acid molar fraction = 0.50:0.50, reaction time t = 7 h, and reaction temperature T = 150 °C. The conversion of the carboxyl group (by titration) was 83.3%, and the maintenance of C=C bonds (by NMR) was 88.7%. Structural characterization confirmed the formation of the desired polymer through FTIR and ^1^H NMR analyses. Molecular weight (*M*_n_ = 1001 g/mol for an optimal product), thermal behavior (DSC, TG, DTG), and rheological properties (*η* = 14.4 and 3.6 Pa∙s for an optimal product at 25 and 36.6 °C) were systematically evaluated. The synthesized POItcSc resins were transparent and exhibited physicochemical properties favorable for extrusion-based 3D printing techniques such as Direct Ink Writing, offering a promising alternative to conventional petrochemical-based inks.

## 1. Introduction

**Figure 1:**
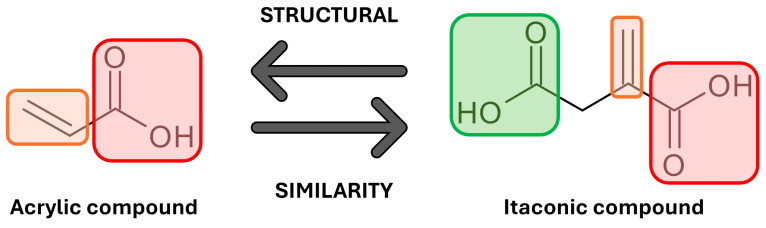
Structural similarity of acrylic and itaconic compounds, where orange refers to the C=C double bond, and green and red to the carboxyl groups.

Most organic molecules are currently synthesized by petrochemical methods [1,2,3,4,5]. However, according to Anastas and Warner’s Green Chemistry principles, renewable materials should be used in syntheses, and toxic solvents and substrates should be limited [4]. Renewable carboxylic acids and their derivatives are commonly used as a source for building blocks with desired properties, to minimize the contribution of toxic materials in industrial applications [3,5]. These primarily include adipic acid, sebacic acid, succinic anhydride, and itaconic acid [6]. These are mainly used in the medical field [3,7]. One of the latest developments is the use of 3D printing in medicine. Three-dimensional printing relies on UV crosslinking of the printed model using mostly toxic and non-renewable acrylic compounds. These are structurally similar to itaconic ones (Figure 1).

**Figure 2:**
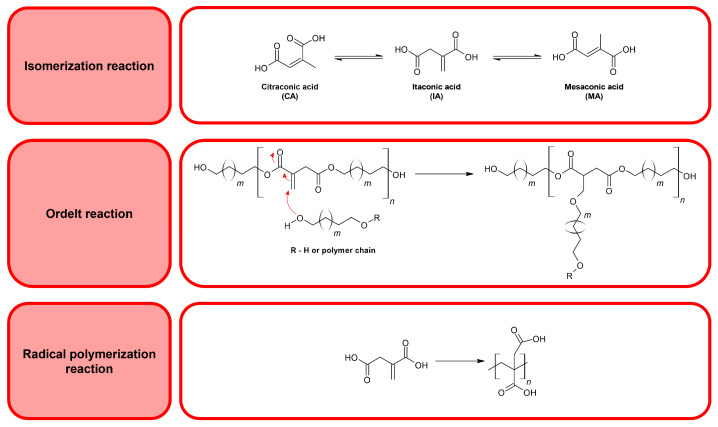
Undesirable side reactions involving itaconic compounds.

Itaconic acid (IA) is an unsaturated dicarboxylic acid obtained from renewable resources [8]. It is commercially produced using an inexpensive method of fermentation of carbohydrates (for instance, glucose, sucrose, and starch) [8,9,10]. It is performed by the *Aspergillus terreus* and *Aspergillus itaconicus* fungi [8,11]. The US Department of Energy describes IA as one of the 12 most valuable organic chemicals [5,12]. This is because of its crucial properties: non-toxicity, biodegradability, and biocompatibility [9]. Furthermore, itaconic compounds exhibit antibacterial and anticancer properties. Because of its advantages, the global demand for IA in 2026 is expected to be more than $110 million [11]. The most crucial feature of itaconic compounds is the existence of a C=C multiple bond in their lateral chain. Its presence makes it possible to perform post-polymerization reactions—that is, the Michael addition or UV-activated photopolymerization reactions [13]. Such a structure of itaconic compounds allows them to be used to synthesize resin-like polymers (for Direct Ink Writing (DIW) 3D printing methods) and produce drugs with anticancer and antiviral properties [14,15,16,17,18]. However, the sterically uncrowded multiple bond can cause problems in synthesizing macromolecular itaconic compounds. During the reaction of obtaining polyesters based on itaconic compounds and compounds having a hydroxyl group in their structure, a Michael reaction can occur (in other words, an oxo-Michael or Ordelt reaction) (Figure 2) [1,19].

Itaconic compounds can isomerize to less reactive isomers: mesaconic and citraconic ones. This, and the radical polymerization reaction of the itaconic unit (Figure 2), are the remaining undesirable reactions of itaconic compounds [17]. The contribution of the isomerization reaction can be reduced by running reactions at temperatures not higher than 150 °C [17]. To reduce the contribution of the undesirable radical polymerization reaction, inhibitors can be incorporated into the reaction system. The most commonly used are 4-methoxyphenol (MEHQ), butylated hydroxytoluene (BHT), phenothiazine, and hydroquinone [20,21,22,23]. However, they are not biocompatible, which makes their use in medicine impossible.

As mentioned, itaconic compounds can be mixed with diols to obtain polyesters. The synthesized aliphatic polyesters exhibit biocompatibility [10]. One such diol is the terminal 1,8-octanediol (1,8-OD). It is the longest water-soluble aliphatic diol [24]. It is commonly used in medicine as a part of medical devices for vascular purposes and to obtain scaffolds [1,25,26,27,28,29,30]. However, as it is challenging to efficiently conduct the synthesis reaction between the itaconic compound and 1,8-octanediol, we decided to enrich the product with succinic anhydride (SAn) [31]. Using saturated succinic anhydride, it is possible to reduce the proportion of the undesirable Ordelt reaction and, consequently, the polycondensation product’s crosslinking degree [32]. Succinic anhydride is a derivative of succinic acid (SA) [6]. It can be produced in the biological fermentation conducted by microorganisms [33]. It is commonly used to obtain biodegradable polymers—for instance, polybutyrate succinate (PBS) and polyamides (Nylon^®^x,4) [33]. As SA and its derivatives are saturated organic compounds, their use makes it possible to extend the polymer chain without the risk of side reactions [34].

In this study, to meet today’s environmental demands, we developed the synthesis of a fully biodegradable and renewable itaconic copolyester—poly(octamethylene itaconate-*co*-succinate) (POItcSc)-copolymer from itaconic acid, succinic anhydride, and 1,8-octanediol, for prospective application in additive manufacturing (similar to poly(butylene adipate-*co*-terephthalate) (PBAT)) [3].

## 2. Materials and Methods

### 2.1. Materials

The following materials were used: itaconic acid (≥99%, Sigma Aldrich, St. Louis, MO, USA), 1,8-octanediol (98%, Angene, Nanjing, China), succinic anhydride (≥99, Gdańsk, Poland), methanol (Chempur, Piekary Śląskie, Poland), 1M aqueous sodium hydroxide solution (Chempur, Piekary Śląskie, Poland), 1M hydrochloric acid solution (Chempur, Piekary Śląskie, Poland), chloroform (Chempur, Piekary Śląskie, Poland), Hanus reagent (Hempur, Piekary Śląskie, Poland), 10% KI solution (Chempur, Piekary Śląskie, Poland), 0.1 M sodium thiosulfate (Chempur, Piekary Śląskie, Poland), starch indicator (Chempur, Piekary Śląskie, Poland), deuterated DMSO (Deuteron GmbH, Kastellaun, Germany), *tert*-butanol, dichloromethane (HPLC grade, Sigma Aldrich, St. Louis, MO, USA), *n*-hexane (POCH, Gliwice, Poland), toluene (Chempur, Piekary Śląskie, Poland), diethyl ether (Chempur, Piekary Śląskie, Poland), ethyl alcohol (POCH, Gliwice, Poland), dichloromethane (POCH, Gliwice, Poland), THF (POCH, Gliwice, Poland), chloroform (Chempur, Piekary Śląskie, Poland), ethyl acetate (POCH, Gliwice, Poland), 1,4-dioxane (POCH, Gliwice, Poland), methanol (Chempur, Piekary Śląskie, Poland), acetone (POCH, Gliwice, Poland), acetonitrile (POCH, Gliwice, Poland), 1-butanol (POCH, Gliwice, Poland), DMF (POCH, Gliwice, Poland), and DMSO (Chempur, Piekary Śląskie, Poland).

### 2.2. Polyester Synthesis

The syntheses were carried out in the Mettler Toledo MultiMax parallel reactor system (Schwerzenbach, Switzerland) in the Hastelloy reactors. The reactants 1,8-octanediol, itaconic acid, and succinic anhydride were used as supplied, without prior preparation.

To obtain the IA fraction to SAn of 0.35, 0.50, and 0.65, the used weight of the reactants was as follows: 1,8-octanediol (22.78 g; 22.38 g; 22.00 g), itaconic acid (7.09 g; 9.96 g; 12.73 g), and succinic anhydride (10.13 g; 7.66 g; 5.27 g). The molar ratio of IA + SAn:1,8-OD was 1:1. The substrates were weighed and placed in the metal and non-transparent reactor, and the used reactants weighed 40.00 g. The reactor was equipped with a mechanical stirrer, a temperature sensor, and a Dean–Stark apparatus.

The reaction procedure was as follows: In the first stage, the reactants were heated for 15 min to *x*_3_ temperature. The temperature was held constant for *x*_2_ h. Then, 30 min after the start of this phase, the reduced pressure was switched on (200 mbar). Finally, the mixture was cooled down to 40 °C for 15 min. The reaction system was stirred throughout the reaction (200 rpm).

### 2.3. Titration Analysis

To calculate the Acid Number (*AN*_tit_) value, about 0.50–1.00 g of the sample was weighed and dissolved in 25.00 mL of methanol. Five drops of indicator, thymol blue, was added to each sample. After dissolution, each sample was titrated with 1 M NaOH_aq_ until the change in color from yellow to blue was observed. The obtained *AN*_tit_ is a mean of three determinations for each sample of POItcSc. A blank test was performed under the same conditions. To calculate the acid number, the following formula was used:

*AN*_tit_ [mg_KOH_/g_sample_] = ((*V* − *V*_0_) × *M*_NaOH_ × 56.1)/*m* (1)

where

*V*—the volume of 1 M NaOH solution used to titrate the investigated sample [cm^3^];

*V*_0_—the volume of 1 M NaOH solution used for blank titration [cm^3^];

*M*_NaOH_—the titer of the solution for the titration (1 M);

56.1—the molar mass of KOH [g/mol];

*m*—the weight of the investigated sample [g].

To calculate the conversion of carboxyl groups (*%conv*_COOH tit_) in the structure of the synthesized product, the following formula was used:

*%conv*_COOH tit_ = (2 × *n*_IA_ − ((*AN*_tit/_1000)/56.1 × *w*)/(2 × *n*_IA_)) × 100% (2)

where

*n*_IA_ —the amount of itaconic acid used in the synthesis [mol];

*w*—the weight of the substrates in the reaction system [g].

To calculate the Ester Number (*EN*_tit_), about 0.20–0.50 g of the sample was weighed and dissolved in a solution of 15.00 mL of methanol and 20.00 mL of 1 M NaOH_aq_. Then, the solutions were refluxed for one hour at around 120 °C. Next, the solution was cooled down to room temperature. Five drops of phenolphthalein were added to each trial. The samples were titrated with 1 M HCl_aq_ until discoloration of the pink solution. A blank test was performed under the same conditions. The obtained *EN*_tit_ is a mean of three determinations for each sample of POItcSc. To calculate the acid number, the following formula was used:

*EN*_tit_ [mg_KOH_/g_sample_] = (((*V*_0_ − *V*) × *M*_HCl_ × 56.1)/*m*) − *AN*_tit_ (3)

where

*V*—the volume of aqueous 1 M HCl solution used to titrate the investigated sample [cm^3^];

*V*_0_—the volume of aqueous 1 M HCl solution used for blank titration [cm^3^];

56.1—the molar mass of KOH [g/mol];

*m*—the weight of the investigated sample [g].

The final result is the average of three determinations.

To calculate the esterification degree (*ED*_tit_), the following formula was used:

*ED*_tit_ [%] = *EN*_tit_/(*EN*_tit_ + *AN*_tit_) × 100% (4)

where

*EN*_tit_—the ester number from titration;

*AN*_tit_—the acid number from titration.

To calculate the Iodine Number (*IN*_tit_), approximately 0.50 g of the investigated sample was weighed and dissolved in a solution of 10.00 mL of chloroform and 15.00 mL of Hanus reagent. After mixing the flask contents for 30 min in a dark place, 15.00 mL of 10% KI solution and 50 mL of distilled water were added. Then, the solution was titrated with 0.1 M sodium thiosulfate solution until a bright orange color was observed. Next, 5 mL of starch indicator was added to the flask to obtain a dark blue color in the mixture. Then, the solution was titrated with 0.1 M thiosulfate solution until it discolored. The obtained *IN*_tit_ is a mean of two determinations for each sample of POItcSc. To calculate the iodine number, the following formula was used:

*IN*_tit_ = 1.269 × ((a − b)/c)) (5)

where

a—the volume of sodium thiosulfate solution (0.1 M) used for blank titration [cm^3^];

b—the volume of sodium thiosulfate solution (0.1 M) used to titrate the sample [cm^3^];

c—the weight of the investigated sample [g].

To calculate the percentage of unreacted C=C double bonds (*%*_C=C IN tit_) in the structure of the POItcSc, the following formula was used:

*%*_C=C IN tit_ = ((((*IN*_tit/_100)/253.81) × *w*)/*n*_IA_) × 100% (6)

where

253.81—molar mass of the molecular iodine (I_2_) [g/mol].

### 2.4. Fourier Transform Infrared (FT-IR) Analysis

FT-IR spectra were recorded using an ALPHA spectrometer (Bruker, Berlin, Germany), ranging from 400 to 4000 cm^−1^, with 32 scans for each sample.

### 2.5. Nuclear Magnetic Resonance (NMR) Analysis

^1^H NMR and ^13^C NMR measurements of the obtained polyesters were performed on an Agilent 400 MHz NMR spectrometer with deuterated dimethyl sulfoxide (DMSO-d_6_) as a solvent and tert-butanol (*t*-BuOH) as the internal chemical shift standard.

### 2.6. Gel Permeation Chromatography (GPC) Analysis

The number-average molecular weight (*M*_n_), weight-average molecular weight (*M*_w_), and dispersity index (*DI*) of the synthesized products were determined by Size Exclusion Chromatography (SEC) carried out on an Agilent 1260 Infinity System (Santa Clara, CA, USA). The setup included an isocratic pump, an autosampler, a degasser, a thermostated column, and a differential refractometer (MDS RI Detector). Data acquisition and analysis were performed using Addon Rev. software (version B.01.02, Agilent Technologies, Santa Clara, CA, USA). *M*_w_ values were calculated based on calibration with linear polystyrene standards (580–128,900 g/mol). The separation process incorporated a pre-column guard (3 μm, 50 × 7.5 mm) along with two analytical columns: PLgel MIXED-D (5 μm, 300 × 7.5 mm) and PLgel MIXED-E (3 μm, 300 × 7.5 mm). The analyses were conducted at 30 °C using dichloromethane (HPLC grade, Sigma Aldrich, St. Louis, MO, USA) as the mobile phase, with a 0.8 mL/min flow rate.

### 2.7. Thermal Analysis

DSC (Differential Scanning Calorimetry) measurements were conducted on the Q2000 DSC analyzer (TA Instruments, Eschborn, Germany). The DSC procedure was as follows. In the first step, the sample (weighing approximately 10 mg) was weighed and placed in the crucibles, which were closed with lids with holes. Then, the chamber was sealed, and the measurement was performed. At first, the sample was cooled to −90 °C. Then, it was heated to 250 °C (10 °C/min step). In the next step, the sample was cooled to −90 °C. In the final stage, the sample was heated again to 250 °C. DSC thermograms were analyzed using TA Instruments Universal Analysis 2000 software. The glass transition temperature was determined as a midpoint temperature. The cold crystallization temperature was defined as the peak temperature. DSC analyses were conducted in the nitrogen flow (50 mL/min).

TG (Thermogravimetry) measurements were conducted on the SDT Q600 analyzer (TA Instruments, Eschborn, Germany). The weight loss of the samples (weighing approximately 10 mg) was analyzed using the temperature range from room temperature to 500 °C (10 °C/min step). TG analyses were conducted in the nitrogen flow (100 mL/min).

### 2.8. Rheological Analysis

The MCR 301 rheometer (Anton Paar, Graz, Austria) was used to measure the viscosity of POItcSc products. The plate–plate method was applied. The diameter of the moving plate was 25 mm. Every experiment lasted for 8 min (4 min/stage). In the first stage, the shear rate increased from 0.01 cm^−1^ to 10 cm^−1^. The shear rate decreased from 10 cm^−1^ to 0.01 cm^−1^ in the second and final stage. The number of measurement points per decade was 20. To approximate the measurement curve, the Casson model was used:

(7) $$\tau_{1/2}\;=\;\tau_{1/2}^{0}\;+\;{\left(\eta_{p}\;\times \;\dot{\gamma }\right)}^{1/2}$$

where

τ—shear stress [Pa];

τ^0^—shear limit (yield stress) [Pa];

*η*_p_—rheological parameter (plastic viscosity) [Pa∙s];

$\dot{\gamma }$—shear rate [s^−1^].

The graphs of the relationship between the tangential stress’s square root and the shear rate’s square root were obtained using the data. For each graph, the regression equation was calculated. The straight line’s directional coefficient was the plastic viscosity’s square root raised to the power of 2. The estimated value was the plastic viscosity.

Amplitude sweep testing of the POItcSc was conducted at 25 °C and 36.6 °C. Three different frequencies were investigated: 0.1 Hz, 1 Hz, and 10 Hz. Each measurement preceded a phase in which the sample was subjected to a constant, lowest strain (γ = 0.005%). Then, measurements were carried out at increasing strain (γ = 100%). The range of linear viscoelasticity of the POItcSc was determined.

Frequency sweep testing of the POItcSc was carried out at 25 °C and 36.6 °C in the range from 0.01 Hz to 100 Hz, with a constant strain of 0.1%. The measurement phase was preceded by a pre-shearing phase, in which the test sample was subjected to sweeping at a continuous frequency of 0.01 Hz for 120 s at a constant strain of 1%.

To determine the dependence of the form modulus (G′) and loss modulus (G″) as a function of temperature, POItcSc was heated at a rate of 3 °C/min from 15 °C to 195 °C, at a constant strain of 0.1% and a constant frequency of 1 Hz.

### 2.9. Viscosity-Visual-Utility (VVU) Analysis

The numerical interval scale characterized every synthesis product from the mathematical model for its specified properties (Table 1) at two temperatures: *T*_room_ and T = 36.6 °C. The numerical interval scale was converted into a percentage scale. The highest number of possible points, 32, was defined as 100%, and the lowest number of possible points, 10, was described as 0%.

### 2.10. Statistical Analysis

All results were analyzed statistically using Statistica software (Version 13.3). Comparisons between groups were performed using one-way ANOVA. Differences were considered statistically significant when *p*-values < 0.05.

## 3. Results and Discussion

**Figure 3:**
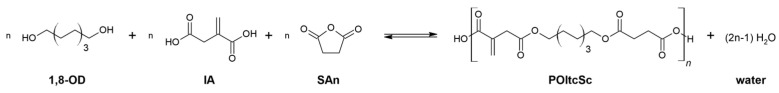
Poly(octamethylene itaconate-*co*-succinate) synthesis.

The main objective of the performed experiments is an in-depth investigation of the poly(octamethylene itaconate-*co*-succinate) synthesis products obtained under different conditions in the polyesterification process [35]. In the research, no catalyst, inhibitor, or solvent was used to obtain the final product. We obtained poly(octamethylene itaconate-*co*-succinate) in the polycondensation reaction between itaconic acid, succinic anhydride, and 1,8-octanediol (Figure 3). The synthesis was performed without the use of a catalyst, as it can cause difficulties in the intended final application and can contribute to the presence of an undesired Ordelt reaction [16,17,36].

### 3.1. Statistical Analysis

To optimize the synthesis of POItcSc, the Box–Behnken mathematical plan was used, as it allows avoiding experiments in extreme conditions (only +1 or −1 values) and reduces research time (fewer experiments) [37]. Fifteen experiments were conducted. Three of those fifteen experiments were conducted under the same conditions to examine the repeatability of the results and the experimenter’s reliability.

The three chosen input variables (*x*) were as follows:

*x*_1_—molar fraction of itaconic acid in the reaction system (IA molar fraction) (primarily for optimization of molecular weight, rheological and thermal properties, transparency and homogeneity of the product, and contribution of side reactions);

*x*_2_—time of the POItcSc synthesis (t), [h] (to define the variability of carboxyl group conversion, the contribution of side reactions, and the consistency of the product);

*x*_3_—temperature of the POItcSc synthesis (T), [°C] (to determine whether it affects the contribution of side reactions and the consistency of the product).

The three output variables (*y*), most relevant regarding the subsequent use of the obtained macromolecular product, were selected. These were, in sequence,

*y*_1_—percentage conversion of carboxyl groups -COOH (*%conv*_COOH tit_) (calculated from the *AN*_tit_), [%];

*y*_2_—percentage of unreacted unsaturated C=C double bonds (*%*_C=C 1H NMR_) (calculated from the ^1^H NMR spectra analysis), [%];

*y*_3_—Viscosity-Visual-Utility analysis (*%*_VVU_), [%].

The selection of these parameters as output variables was clarified in a previous article [37].

**Figure 4:**
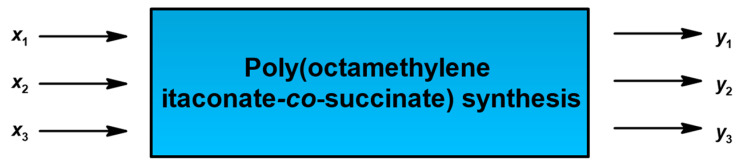
“Black box” of the poly(octamethylene itaconate-*co*-succinate) synthesis.

To facilitate the understanding of the use of input/output variables, the POItcSc synthesis model is presented as a “black box” in Figure 4.

Table S1 presents the coded values of the input variables used in the Box–Behnken plan. A temperature of 150 °C was selected as the maximum temperature for POItcSc syntheses to minimize the contribution of undesirable side reactions [38,39,40,41]. A summary of the coded values of the input variables and the experimental and calculated values of the output variables is presented in Table 2.

According to the NMR (Nuclear Magnetic Resonance) analysis results (Table S2), the contribution of the mesaconic compound in the reaction system is mild (from 0.5 to 3.7%), thanks to the use of a synthesis temperature no higher than 150 °C [16,21]. However, not all received products had the desired consistency (Figure S1). They were in the form of wax, which resulted from a synthesis temperature that was too low and a low value of the esterification degree (Table S2). It should be noted that a slight increase in the temperature of the sample (to 36.6 °C) contributed to obtaining a product with the desired resin consistency. This, and the results of the viscosity (Table S2) for some of the products (9, 11, 12, 14, 15), lead to the conclusion that POItcSc is a suitable macromolecular compound for use as, for instance, 3D printing ink in the DIW method.

Every product obtained in the conducted experiments was investigated for its molecular weight by GPC-SEC (Gel Permeation Chromatography-Size Exclusion Chromatography) experiments (Table S2). The resultant elugrams are usually used for polycondensation products (Gaussian distribution) [41]. The obtained *M*_w_ (weight-average molecular weight) of the products is in the range of 821 to 3370 (g/mol), which contributes to the number of repeating units from 4 to 16 (assuming that the repeating unit consists of one itaconate, succinate, and diol unit). The dispersity index (*DI*) ranges from 1.7 to 4.0, which is anticipated for the polycondensation reactions [42]. Compared with other macromolecular compounds with itaconate segments for additive manufacturing purposes, the obtained POItcSc is characterized by similar values of molecular weights determined by GPC analyses [1,42,43,44,45]. Structural differences cause the main differences in the assigned values.

To define which regression equation coefficients are significant, the *t*-Student test was used (∣*t*_calculated_∣ > *t*_critical_). The probability level (*p*-value) was 5%, and the *t*_critical_ = 4.303. The *F*-Snedecor test (*F*_critical_ (0.05; 3; 2) = 19.16) was performed to determine the adequacy of the regression equations.

A Pareto Chart (Figures S3–S5) was presented as a graphical representation for the Analysis of Variance (ANOVA) for every output variable. Below, all regression equations, which describe every output variable, are presented (where the green color corresponds to the relevant coefficients):

*y*_1_ = **73.4** − **8.56** × *x*_1_ + **11.1** × *x*_2_ + **9.97** × *x*_3_ + **3.95** × *x*_1_ × *x*_2_ + 0.184 × *x*_1_ × *x*_3_ + 0.398 × *x*_2_ × *x*_3_ + 1.51 × *x*_1_^2^ − 0.603 × *x*_2_^2^ + 9.97 × *x*_3_^2^ (8)

*y*_2_ = **79.2** − 0.743 × *x*_1_ − 1.14 × *x*_2_ + **6.53** × *x*_3_ + 0.558 × *x*_1_ × *x*_2_ − 2.30 × *x*_1_ × *x*_3_ + 4.13 × *x*_2_ × *x*_3_ − 4.31 × *x*_1_^2^ − 2.36 × *x*_2_^2^ + 0.103 × *x*_3_^2^ (9)

*y*_3_ = **80.2** + **5.47** × *x*_1_ + 0.391 × *x*_2_ + 0.391 × *x*_3_ + **3.13** × *x*_1_ × *x*_2_ − 1.56 × *x*_1_ × *x*_3_ + 0.781 × *x*_2_ × *x*_3_ − **3.00** × *x*_1_^2^ + 4.04 × *x*_2_^2^ − **5.34** × *x*_3_^2^ (10)

**Figure 5:**
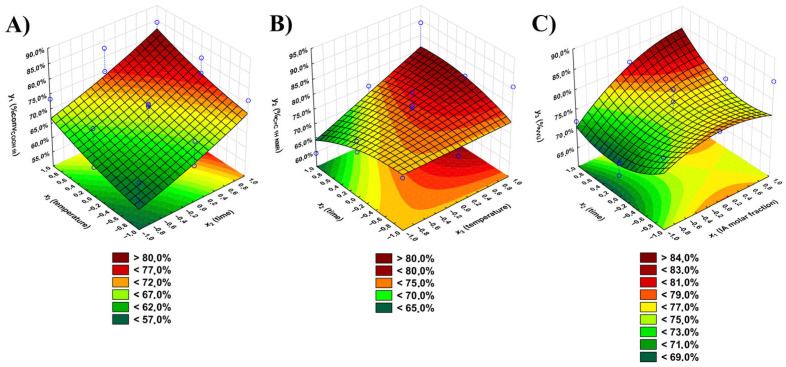
Dependence of the (**A**) percentage conversion of carboxyl groups -COOH of the POItcSc product on the reaction’s temperature (*x*_3_) and time (*x*_2_), *x*_1_ = 1; (**B**) percentage of unreacted C=C double bonds of the POItcSc product on the reaction’s time (*x*_2_) and temperature (*x*_3_), *x*_1_ = 1; (**C**) Viscosity-Visual-Utility analysis of the POItcSc product on the reaction’s time (*x*_2_) and IA molar fraction (*x*_1_); *x*_3_ = 1. The blue circles correspond to the conditions of the performed reactions.

The graphical representations of the above regression equations are shown in Figure 5.

For the *y*_1_ variable, every input variable is significant (∣*t*_calculated *x*1_∣ = 8.31; (∣*t*_calculated *x*2_∣ = 10.80; (∣*t*_calculated *x*3_∣ = 9.68), as is the linear relationship between the *x*_1_ and *x*_2_ (∣*t*_calculated *x*1*x*2_∣ = 2.71) input variables. However, the inclusion of other investigated variables, their powers, and their relations in the model increase the coefficient of determination R^2^ value from 0.96 to 0.98. This means that approximately 98% of the variability of the %*conv*_COOH tit_ originates from the variability of the investigated input variables. As the *F*_calculated_ for *y*_1_ (17.66) variable is smaller than *F*_critical_, the regression equation for the *y*_1_ variable can be defined as sufficient. The experimental and calculated values of the *y*_1_ variable differ only by ±4.1 percentage points (Table 2), which shows the perfect fit for the used model. Reaction time and temperature play a significant role in the conversion of carboxyl groups. The longer the reaction time, and with higher temperature, the greater the %*conv*_COOH_ (>80%).

For the *y*_2_ variable, only the *x*_3_ input variable plays a significant role (∣*t*_calculated *x*3_∣ = 3.14) in the regression equation. Using only this coefficient for the model, the coefficient of determination R^2^ has a low value (0.48). This means many other factors not included in the research affect the *%*_C=C 1H NMR_ value (for instance, pressure in the reaction system or factors beyond the researcher’s control). The inclusion of insignificant coefficients increases the R^2^ value to 0.75. The *F*_calculated_ for the *y*_2_ variable is 6.80, meaning there is no evidence to reject the hypothesis that the presented equation is adequate. However, it should be noted that other factors affecting the value of the *y*_2_ variable were not considered. Similar to the *%conv*_COOH tit_ values, temperature has the most crucial role. Although side reactions involving multiple bonds are expected to occur more frequently at higher temperatures, the opposite trend was observed. This may be due to the use of two acidic monomers (itaconic acid and succinic anhydride), which contributed to the reduction of the unfavorable effect of temperature on the occurrence of reactions by multiple bonds of the itaconic unit. This was confirmed by the result of the *t*-Student test, in which the power of the input variable *x*_1_ shows almost the highest significance, right after the variable *x*_3_. The experimental and calculated values of the *y*_2_ variable differ only by ±5.3 percentage points (Table 1), which represents a perfect fit for the used model.

For the *y*_3_ variable, the IA molar fraction (∣*t*_calculated *x*1_∣ = 9.30), squared temperature (∣*t*_calculated *x*3_^2^∣ = 6.17), time (∣*t*_calculated *x*2_^2^∣ = 4.66), IA molar fraction (∣*t*_calculated *x*1_^2^∣ = 3.46), and IA molar fraction with time linear relationship (∣*t*_calculated *x*1*x*2_∣ = 3.76) have a crucial role in obtaining a product with desired properties for 3D printing. Because the incorporation of other coefficients into the model increases the R^2^ value (from 0.94 to 0.97), they were included in the model. For the *y*_3_ variable, *F*_calculated_ = 0.75, meaning that there is no evidence to reject the hypothesis that the equation is adequate. The experimental and calculated values of the *y*_3_ variable differ only from -2.1 to 1.2 percentage points (Table 2), which indicates a perfect fit for the applied model. The product with the highest *%*_VVU_ can be obtained in the reaction with the highest IA molar fraction to the SAn (0.65:0.35), using the longest examined reaction time (t = 7 h). Then, the *%*_VVU_ can exceed 85.0%. The higher the IA molar fraction, the higher the *%*_VVU_ values.

The least squares method (Statistica software) was used to specify the optimal conditions of the POItcSc synthesis. For this, the utility profile function software was used (Figure S6), and the output variables were programmed as variables with low, medium, and high utility (Table S3). To obtain a product with the highest values of investigated output variables (Figure S7), the reaction has to be performed under the following conditions: IA molar fraction 0.50:0.50 (*x*_1_), reaction time = 7 h (*x*_2_), and temperature = 150 °C (*x*_3_). The output variables’ values for the optimal conditions of the product are shown in Table 3.

Considering that the experimental and determined values of the output variables vary negligibly, and the model’s utility is 84.1%, a remarkable fit is demonstrated between the statistical model and reality. The optimal product is characterized by one of the higher molecular weights obtained in the performed experiments—the *M*_w_ was 3129 g/mol, with a dispersity index of 3.1. More extensive product characteristics are shown in Table S2.

### 3.2. FT-IR Spectroscopy and NMR Analysis

**Figure 6:**
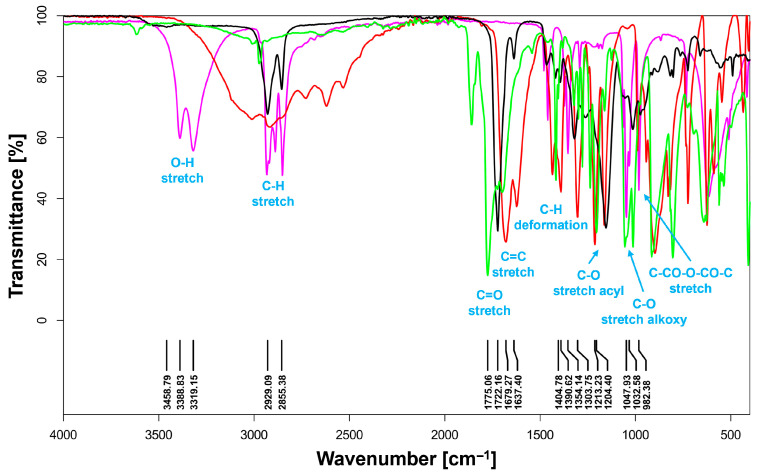
FTIR spectra of poly(octamethylene itaconate-*co*-succinate) (**black line**), itaconic acid (**red line**), 1,8-octanediol (**pink line**), and succinic anhydride (**green line**).

**Figure 7:**
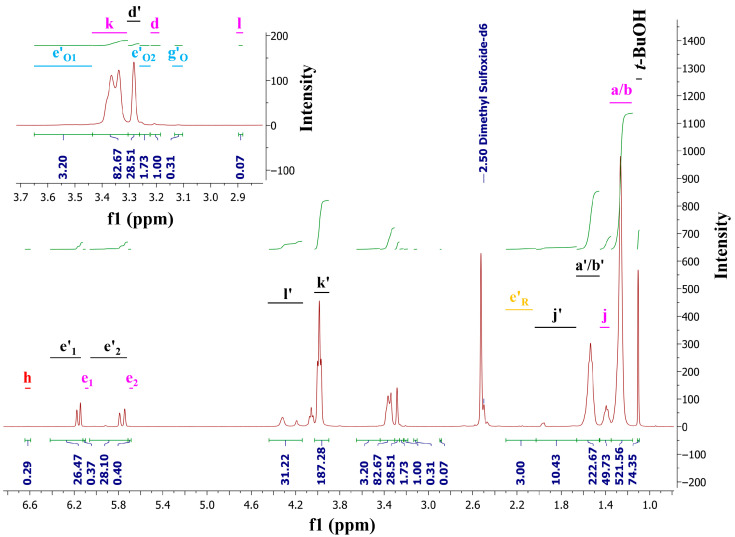
^1^H NMR spectra of poly(octamethylene itaconate-*co*-succinate).

The structure of the substrates and POItcSc product was confirmed by FT-IR (Fourier Transform Infrared) (Figure 6), ^1^H NMR (Figure 7), and ^13^C-NOE NMR (Figure S8).

The O-H bond stretching vibrations can be seen in the 3550–3000 cm^−1^ range. These correspond to the substrates—1,8-octanediol (strong and sharp signal) and itaconic acid (strong and wide signal). For the POItcSc product, there is no visible signal from the O-H group, meaning that the product is not mostly terminated with diol. Furthermore, only a small proportion of unreacted substrates are present in the final product. The 29400–2850 cm^−1^ bands correspond to the methylene groups’ stretching vibrations. The most important signals can be observed in the range below 2000 cm^−1^. The bands in the 1780–1715 cm^−1^ range correspond to the stretching vibrations of the carbonyl groups from α and β-unsaturated esters. In the 1680–1620 cm^−1^ range, stretching vibrations of the C=C double bond can be seen. The presence of polyester can be confirmed by the signals in the range of 1220–1140 cm^−1^ (stretching vibrations of the acyl groups) and 1050–1030 cm^−1^ (stretching vibrations of the acyl groups). In the 1000–980 cm^−1^ range, a signal corresponds to the C-CO-O-CO-C stretching vibrations from the substrate, succinic anhydride.

The ^1^H NMR (Figure 7) was used in calculations (see Supporting Information). Figure S9 presents the assignment of H and C atoms to the signals on NMR spectra.

In each obtained spectra, the characteristic resonance signals corresponding to the C=CH_2_ protons of itaconic acid in the polycondensation products can be observed at 6.1–6.4 ppm (e′_1_) and 5.7–6.1 ppm (e′_2_). This means that there are unreacted double bonds, which are available for subsequent photo-crosslinking. The signals corresponding to the mesaconic isomer are visible on every ^1^H NMR. However, they are weak, and the contribution of the mesaconic isomer is in the range of 0.5% to 3.7%. Signals that correspond to the protons corresponding to the occurrence of undesired radical polymerization (in the range of 2.4% to 8.8%) and Ordelt reactions (in the range of 8.4% to 34.4%) were also observed.

Similar to the ^1^H NMR spectra, the ^13^C NMR-NOE spectra (Figure S8) confirm the successful polycondensation reaction between IA, SAn, and 1,8-OD. The presence of the signals from the carbon atoms corresponding to the C=C double bonds in the polycondensation product (signal E′) confirm that the double bonds of IA were not significantly affected by side reactions.

### 3.3. Thermal Analysis

**Figure 8:**
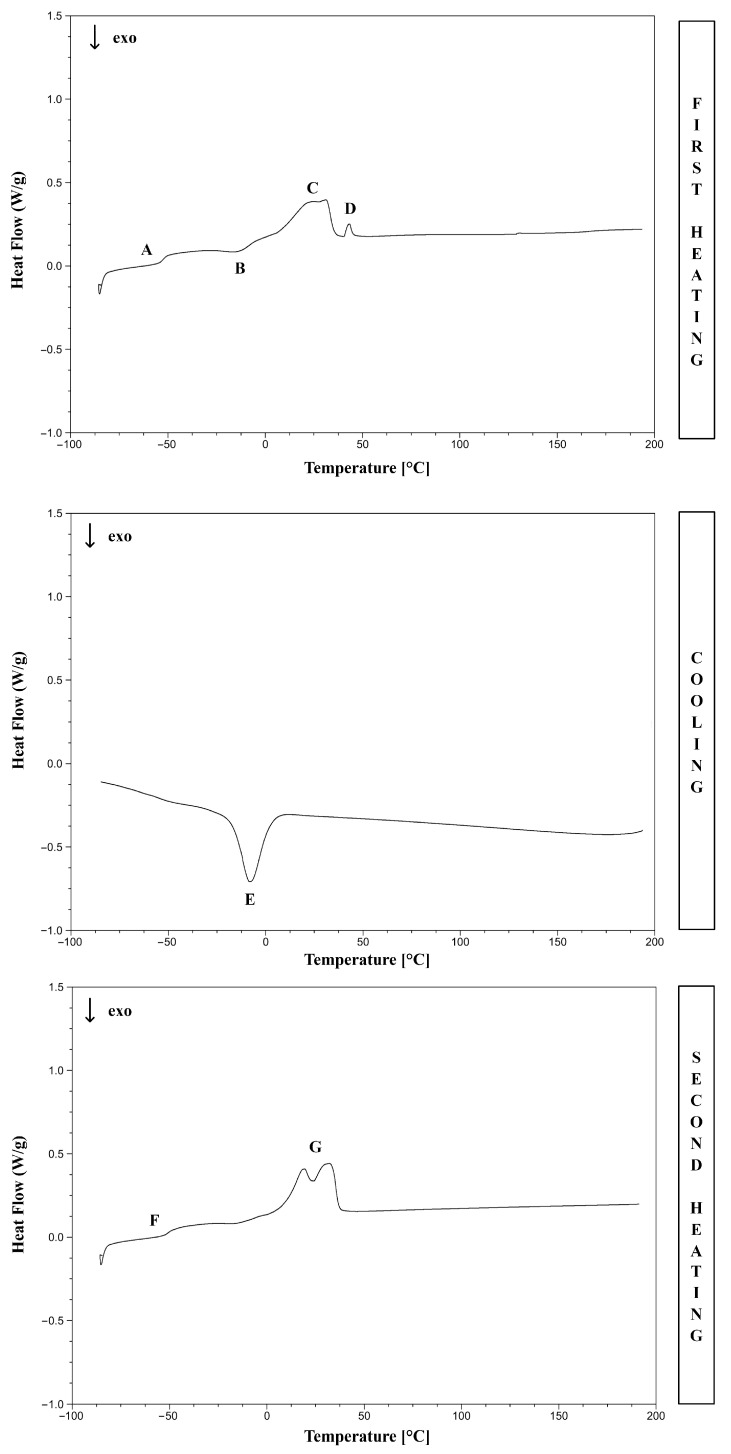
DSC for POItcSc.

DSC (Differential Scanning Calorimetry) was used to examine the thermal properties of the synthesized poly(octamethylene itaconate-co-succinate). Figure 8 shows the characteristic thermal transitions for the POItcSc polymer.

Figure 8 shows some characteristic temperature transitions for POItcSc (Table S6). The glass transition temperature of POItcSc can be seen during the first (*T*_gh1_, A) and second (*T*_gh2_, F) heating cycle. Furthermore, the cold crystallization temperature (*T*_cch1_, B), crystallization temperature (*T*_cc1_, E), and melting temperature (*T*_mh1_, C and *T*_mh2_, G) of POItcSc can also be observed. The determined *T*_g_ values are lower than for the previously characterized poly(tetramethylene itaconate) (PBItc) [38]. Using a diol with a greater proportion of -CH_2_- groups contributes to higher mobility of POItcSc chains [42,46]. The presence of *T*_cc_ and *T*_m_ indicates a semicrystalline polymer nature, mainly due to the use of 1,8-OD. The presence of two endothermic peaks labeled as C indicates the melting of the crystalline phase of POItcSc fractions of different molecular weights. The presence of POItcSc fractions with varying chain lengths was confirmed by GPC analysis, where *DI* = 3.1. An endothermic peak D is also visible during the first heating cycle, corresponding to the melting of unreacted 1,8-OD. The absence of such a peak during the second heating cycle indicates that 1,8-OD underwent the reaction during the first heating cycle.

**Figure 9:**
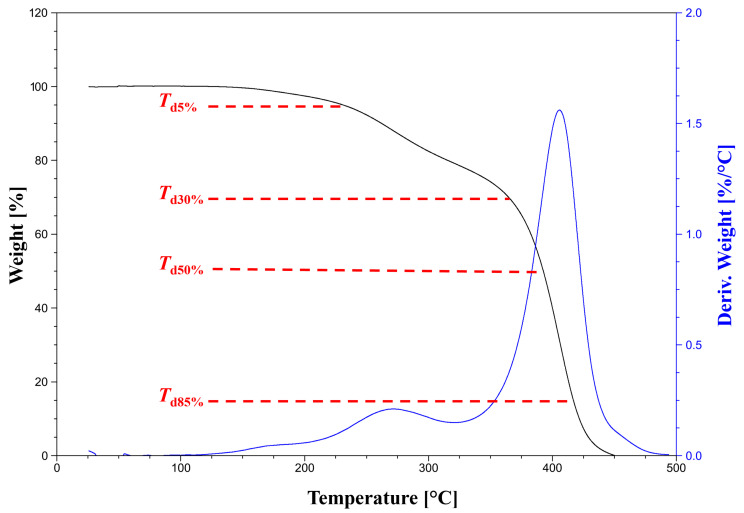
Thermogravimetric degradation and derivative thermogravimetric curve for poly(1.8-octanediol itaconate-*co*-succinate).

The thermal stability of the POItcSc polymer was analyzed by thermogravimetric analysis (TG) (Figure 9).

The characteristic degradation temperatures are summarized in Table S6. As shown on the DTG (Derivative Thermogravimetry) curve, POItcSc exhibits two-stage weight loss, similar to other macromolecular compounds within itaconate units [47]. A minor weight loss at about 100 °C is due to water removal from the reaction system [48]. The first significant mass loss occurs at about 270 °C, which is associated with the degradation of intermediate decay products [48]. The maximum weight loss is observed at 405.5 °C (*T*_d max_). At this temperature, the dissociation of ester bonds occurs [49,50,51]. Then, the dehydrogenation and dissociation of ashes formed at lower temperatures occur [52]. Such high degradation temperatures indicate that a polymer with high thermal stability has been synthesized [53]. Furthermore, the obtained POItcSc polycondensation product shows higher thermal stability than other previously synthesized itaconate macromolecular compounds, namely BItc [38]. This is due to the use of a longer aliphatic diol than 1,4-butanediol. As the thermal stability is higher, the POItcSc final product for application will tend to maintain its physical and mechanical properties at high temperatures for a longer time than PBItc [54].

Considering the applicability of the obtained POItcSc resin, besides the possibility of its use as an ink for 3D printing by DIW or DLP (Digital Light Processing), based on thermal analysis, it is possible to consider it for obtaining thermoplastic polyester films. This is due to a low melting temperature range, rapid crystallization, and high thermal stability, which suggests good processability [55].

### 3.4. Rheology Analysis

**Figure 10:**
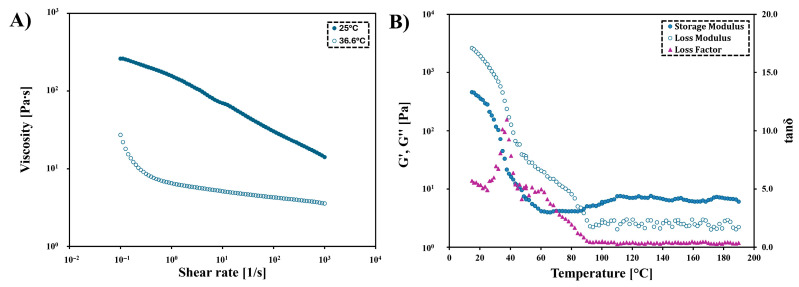
Continuous flow tests of POItcSc at a shear rate equivalent to those experienced during 3D printing at 25 °C and 36.6 °C (**A**), and temperature dependence of the storage modulus, loss modulus, and loss factor for POItcSc (**B**).

The resin produced in the optimal conditions was analyzed for its rheological properties. A shear-thinning product was obtained (Figure 10A) because the product’s viscosity decreases as the shear rate increases. As the shear rate increases, the polymer aggregates disintegrate, and the polymer chains orient and align parallel to the shear direction. From this, it can be specified that the received resin will be suitable for 3D printing with DIW methods, for example. It will easily pass through the printer’s nozzle under pressure, and once it is placed on the table, it will retain its shape. Furthermore, with the higher measuring temperature, the viscosity of the investigated macromolecular product decreases (at 25.0 °C = 14.4 Pa∙s, at 36.6 °C = 3.6 Pa∙s). As the temperature rises, the movement of the polymer chains is facilitated. Thus, the obtained product can again be considered suitable for applications requiring material injection molding, e.g., for DIW 3D printing methods. The obtained product has higher viscosity than the other resins with itaconic fragments (*η* = 0.27–1.66 Pa∙s) [42]. Its reduced viscosity was due to the use of diluents. In the case of POItcSc, its addition was avoided because of the toxicity of those most commonly used ones [42,56]. In the case of PIDDOL (poly(ester thioether) based on 1,12-dodecanediyl bis(methyl itaconate) and linalol), a slightly higher viscosity value for the same measurement conditions was found (*η* = 16.7 ± 1.8 Pa∙s), which was the effect of the use of somewhat longer diol-1,12-dodecanodiol [44].

Thermorheological analysis of POItcSc (Figure 10B) confirmed that the glass transition is observed at a temperature lower than the minimum range of the used measurement apparatus. The intersection of the curves corresponding to the elastic modulus (G′) and loss modulus (G″) occurs at 86.2 °C. The material transitions to an elastic (flexible) state at this temperature. Then, the crosslinking process of POItcSc begins. Considering the potential application of the obtained POItcSc resin, it should be noted that when printing with it, the head temperature must not be greater than the limiting temperature of 86.2 °C.

**Figure 11:**
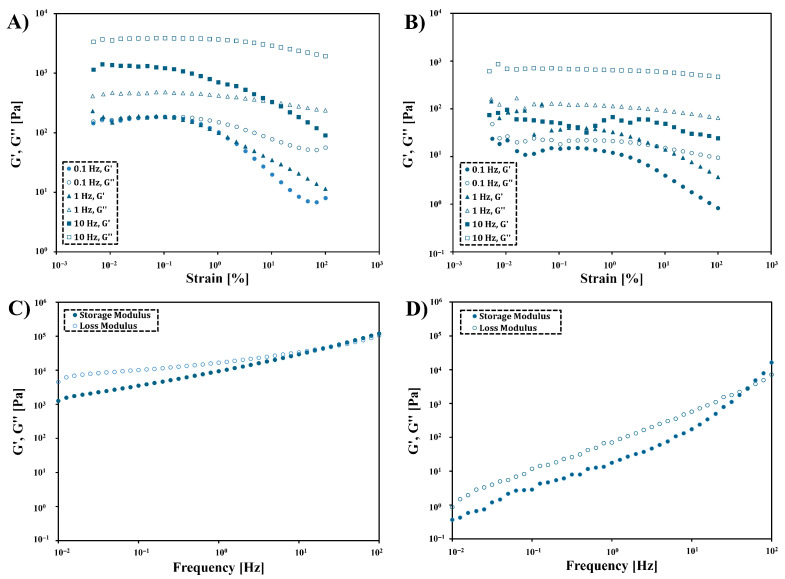
Amplitude sweep of POItcSc at 25 °C (**A**) and 36.6 °C (**B**) and frequency sweep of POItcSc at 25 °C (**C**) and 36.6 °C (**D**).

We performed amplitude sweep tests to determine the range of linear viscoelasticity (LVE) for the obtained POItcSc (Figure 11A,B). Regardless of the experimental temperature, there is an oscillation of G′ and G″ values for low strain values. This indicates the occurrence of the reorganization of polymer chains. With higher temperatures, it occurs more easily. For the tested strain range, the value of G′ < G″ indicates that the viscous values of POItcSc are dominant (compared to elastic properties) [57]. Such behavior is desirable for DIW 3D-printed materials. For the tested product, the range of LVE is present for a strain of 0.1%.

A frequency sweep study was conducted for POItcSc (Figure 11C,D). Frequency sweep tests confirmed that viscous properties dominate over elastic properties (G′ < G″) for POItcSc. This indicates that a non-crosslinked or low-crosslinked product was obtained. The intersection of the curves occurs at the higher measurement temperature (36.6). In other words, in the case of 3D printing POItcSc resin with a heated printer extruder, the resin would pass through the printer nozzle more easily and hold its shape better immediately after printing. This means that carrying out 3D printing of POItcSc at an elevated temperature is beneficial.

## 4. Conclusions

We successfully synthesized a set of biobased poly(octamethylene itaconate-*co*-succinate) polyesters from itaconic acid, 1,8-octanediol, and succinic anhydride in a catalyst-free and solvent-free melt polycondensation reaction. For this, we used the Box–Behnken mathematical model to optimize the synthesis of POItcSc. We determined how the itaconic acid molar fraction in combination with succinic anhydride, the reaction time, and temperature influence the properties of the obtained polycondensation product. The results show that the presence of succinic anhydride as a co-monomer reduced the percentage of the undesired side reactions. The *M*_n_ of the obtained products was defined using GPC. Every product was examined for its rheological properties. The product obtained in optimal conditions was additionally characterized using DSC, TG (DTG), and rheological (oscillation) methods. The performed statistical analysis revealed that to receive a product with the best properties in terms of potential use, the reaction should be conducted under the following conditions: IA molar ratio = 0.50:0.50, reaction time = 7 h, and reaction temperature T = 150 °C. The optimal POItcSc product is a semi-crystalline polyester with proper viscosity and consistency (resin) for 3D printing.

As for now, POItcSc may be considered as a potential ink for 3D printing purposes (for instance, in medicine). However, extensive studies on the UV-crosslinking of POItcSc and the properties of the UV-crosslinked films need to be performed in the future.

## Figures and Tables

**Table 1:** Viscosity-Visual-Utility evaluation for the obtained POItcSc products.

Structure		Consistency		Transparency		Ability to Spread the Sample on the Table		Viscosity [Pa∙s]	
1	Hard and brittle	1	Wax	1	None	1	Yes	1	*η* < 10 or *η* > 1000
2	Incompressible and sticky	2	Wax/Resin	2	Partial	2	Partial	2	500 < *η* < 1000
3	Compressible and sticky	3	Resin	3	Full	3	No	3	100 < *η* < 500
								4	10 < *η* < 100

**Table 2:** Experimental matrix and the calculated results for the output variables.

No.	Coded Variable			*%conv*_COOH tit [%]_			*%*_C=C 1H NMR_ [%]			*%*_vvu_ [%]		
	*x*_1_	*x*_2_	*x*_3_	Exp. ^1^	Calc. ^2^	Diff. ^3^	Exp.	Calc.	Diff.	Exp.	Calc.	Diff.
**1**	−1	−1	0	60.2	63.5	−3.3	70.9	73.3	−2.4	78.1	78.5	−0.4
**2**	1	−1	0	73.3	70.5	2.7	73.7	73.7	0.0	84.4	83.2	1.2
**3**	−1	1	0	81.0	83.7	−2.7	70.0	69.9	0.0	71.9	73.0	−1.2
**4**	1	1	0	75.9	72.6	3.3	74.9	72.5	2.4	90.6	90.2	0.4
**5**	−1	0	−1	70.8	68.9	1.9	70.5	65.3	5.3	65.6	64.5	1.2
**6**	1	0	−1	62.6	66.7	−4.1	74.3	71.4	2.9	78.1	78.5	−0.4
**7**	−1	0	1	82.7	78.7	4.1	80.0	82.9	−2.9	68.8	68.4	0.4
**8**	1	0	1	74.9	76.8	−1.9	74.5	79.8	−5.3	75.0	76.2	−1.2
**9**	0	−1	−1	65.9	64.5	1.3	72.7	75.5	−2.9	78.1	78.9	−0.8
**10**	0	1	−1	76.0	75.2	0.8	59.7	65.0	−5.3	78.1	78.1	0.0
**11**	0	−1	1	73.3	74.1	−0.8	85.7	80.3	5.3	78.1	78.1	0.0
**12**	0	1	1	84.3	85.6	−1.3	89.2	86.3	2.9	81.3	80.5	0.8
**13**	0	0	0	72.4	73.5	−1.1	77.9	79.0	−1.2	81.3	80.2	1.0
**14**	0	0	0	74.8	73.5	1.4	77.1	79.0	−2.0	78.1	80.2	−2.1
**15**	0	0	0	73.2	73.5	−0.3	82.2	79.0	3.2	81.3	80.2	1.0

^1^ Exp.—Experimental; ^2^ Calc.—Calculated; ^3^ Diff.—Difference.

**Table 3:** Calculated and experimental results of the output variables obtained for the product under optimal conditions.

Result	*%conv*_COOH tit_ [%]	*%*_C=C 1H NMR_ [%]	*%*_VVU_ [%]
**Calculated**	85.6	86.3	80.5
**Experimental**	83.3	88.7	87.5

## Data Availability

Data are contained within the article.

## Supplementary Materials

The following supporting information can be downloaded at: https://www.mdpi.com/article/10.3390/polym17162220/s1, Figure S1: Consistency of the obtained POItcSc products at 25 °C; Figure S2: Consistency of the obtained POItcSc products at 36.6 °C; Figure S3: Pareto Chart of Standardized Effects for the %*conv*_COOH tit_ (y1) variable (the red line refers to the limit, beyond which the coefficient of the regression equation becomes significant); Figure S4: Pareto Chart of Standardized Effects for the *%*_C=C 1H NMR_ (*y*_2_) variable; Figure S5: Pareto Chart of Standardized Effects for the %_VVU_ (*y*_3_) variable; Figure S6: Profile of the approximated values of input variables and utility of the used mathematical model; Figure S7: Consistency of the optimal product; Figure S8: ^13^C NMR spectra of poly(octamethylene itaconate-*co*-succinate); Figure S9: Assignment of protons and carbon atoms to the corresponding signals on the ^1^H NMR and ^13^C NMR spectra; Table S1: Coded input variables in the Box–Behnken plan for the PISO synthesis optimization; Table S2: Characterization of the polycondensation products; Table S3: Values of the output variables to generate the response utility profile; Table S4: Significance test of regression equation coefficients for the investigated output variables (numbers are written to four significant digits); Table S5: Model adequacy test for the investigated output variables; Table S6: Thermal analysis of POItcSc polycondensation product.

## Abbreviations

The following abbreviations are used in this manuscript:

*%conv*_COOH tit_	Conversion of carboxyl groups (by titration)
*%*_C=C IN tit_	Percentage of unreacted C=C double bonds (by titration)
*%*_C=C 1H NMR_	Percentage of unreacted C=C double bonds (by 1H NMR)
1,8-OD	1,8-octanediol
ANOVA	Analysis of variance
*AN*_tit_	Acid number (by titration)
BHT	Butylated hydroxytoluene
CA	Citraconic acid
DI	Dispersity index
DIW	Direct ink writing
DLP	Digital light processing
DMSO-d6	Dimethyl sulfoxide
DSC	Differential scanning calorimetry
DTG	Derivative thermogravimetry
*ED*_tit_	Esterification degree (by titration)
*ED*_NMR_	Esterification degree (by NMR)
*EN*_tit_	Ester number (by titration)
FT-IR	Fourier transform infrared
G′	Elastic modulus
G″	Loss modulus
GPC	Gel permeation chromatography
IA	Itaconic acid
*IN*_tit_	Iodine number (by titration)
LVE	Linear viscoelasticity
MA	Mesaconic acid
MEHQ	4-metoxyphenol
*M*_n_	Number-average molecular weight
*M*_w_	Weight-average molecular weight
NMR	Nuclear magnetic resonance
PBAT	poly(butylene adipate-*co*-terephthalate)
PBItc	Poly(tetramethylene itaconate)
PBS	Polybutyrate succinate
PIDDOL	poly(ester thioether) based on 1,12-dodecanediyl bis(methyl itaconate) and linalool
POItcSc	Poly(octamethylene itaconate-*co*-succinate)
SA	Succinic acid
SAn	Succinic anhydride
SEC	Size exclusion chromatography
*t-*BuOH	Tert-butanol
TG	Thermogravimetry
*T*_cc1_	Crystallization temperature
*T*_cch1_	Cold crystallization temperature during the first heating
*T*_d5%_	5% decomposition temperature
*T*_d30%_	30% decomposition temperature
*T*_d50%_	50% decomposition temperature
*T*_d85%_	85% decomposition temperature
*T*_gh1_	Glass transition temperature during the first heating
*T*_gh2_	Glass transition temperature during second heating
*T*_mh1_	Melting temperature during first heating
*T*_mh2_	Melting temperature during second heating
VVU	Viscosity-Visual-Utility
