# Supplementary material for: Towards Greener Polymers: Poly(octamethylene itaconate-co-succinate) Synthesis Parameters
Source: Polymers (Basel). 2025 Aug 14;17(16):2220. doi: 10.3390/polym17162220 (PMC12390383; doi:10.3390/polym17162220)
Supplement: Supplementary file 1 [file polymers-17-02220-s001.zip › polymers-3786998-supplementary.pdf]

Towards Greener Polymers: Harnessing Design of Experiments for Poly(Octamethylene Itaconate-*co*-Succinate)

Magdalena Miętus[1], Tomasz Gołofit[1], Agnieszka Gadomska-Gajadhur[1]

[1]Faculty of Chemistry, Warsaw University of Technology, Noakowskiego 3 Street, 00-664 Warsaw, Poland; magdalena.mietus.dokt@pw.edu.pl (M.M.), tomasz.golofit@pw.edu.pl (T.G.)

*Correspondence: agnieszka.gajadhur@pw.edu.pl (A.G.-G.)

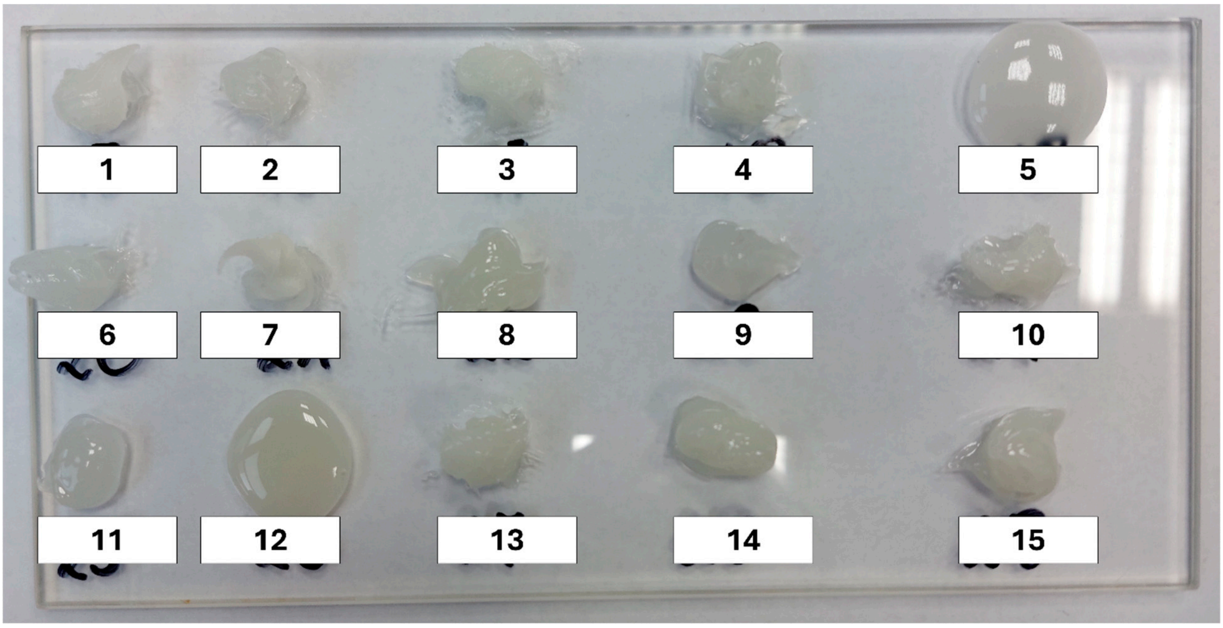

Figure S1. Consistency of the obtained POItcSc products at 25°C.

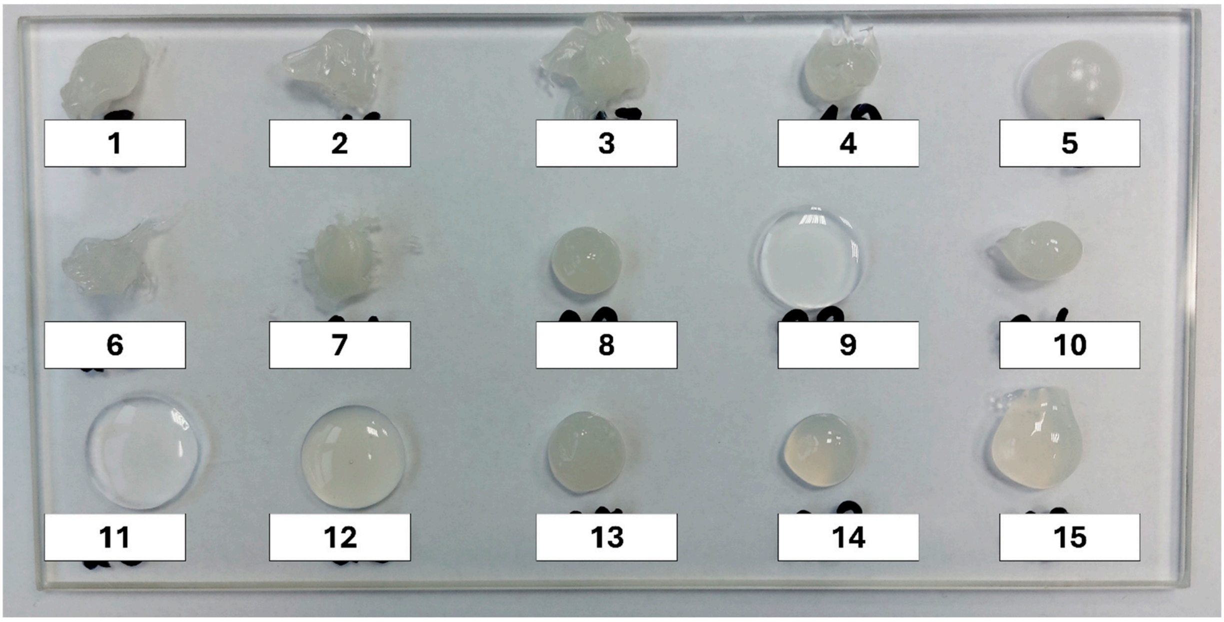

Figure S2. Consistency of the obtained POItcSc products at 36.6°C.

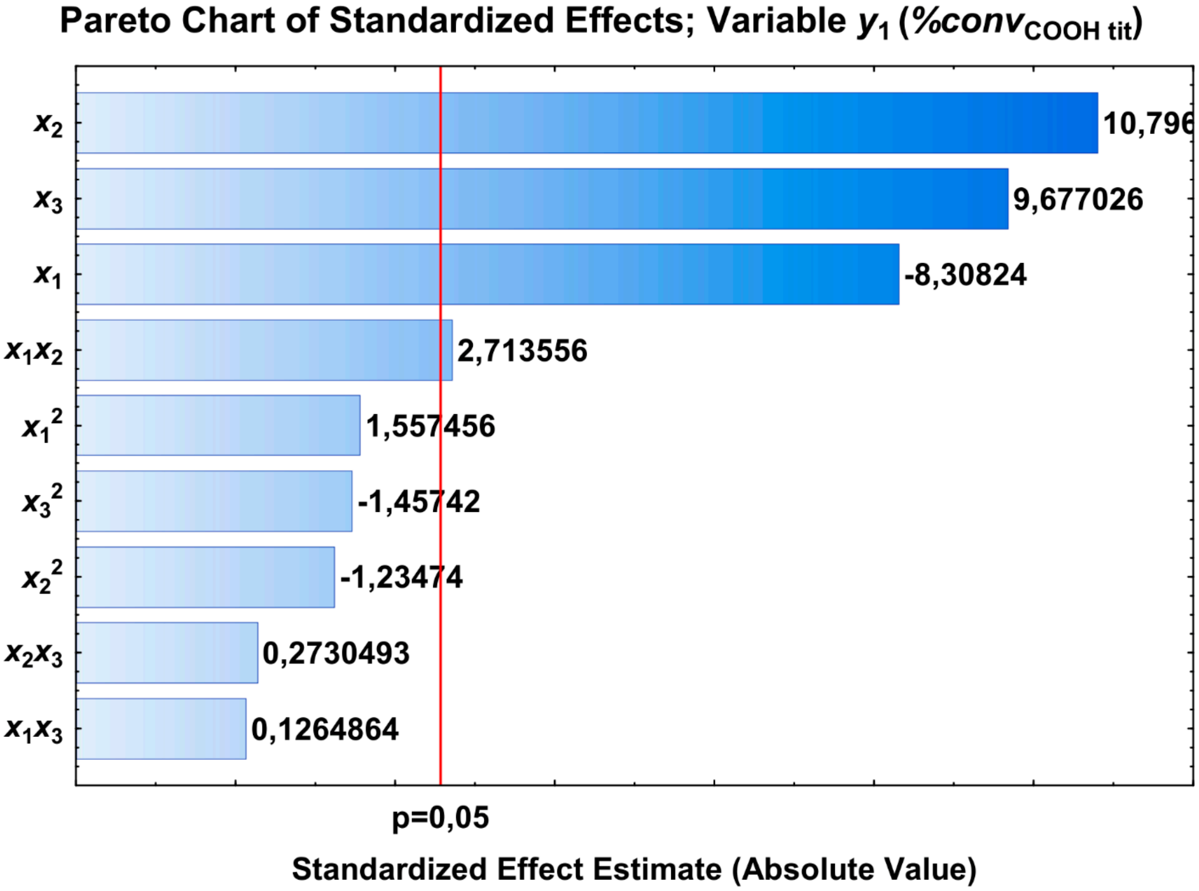

Figure S3. Pareto Chart of Standardized Effects for the *%conv*$_{\mathrm{COOH\ tit}}$ (y_1) variable (the red line refers to the limit, beyond which the coefficient of the regression equation becomes significant).

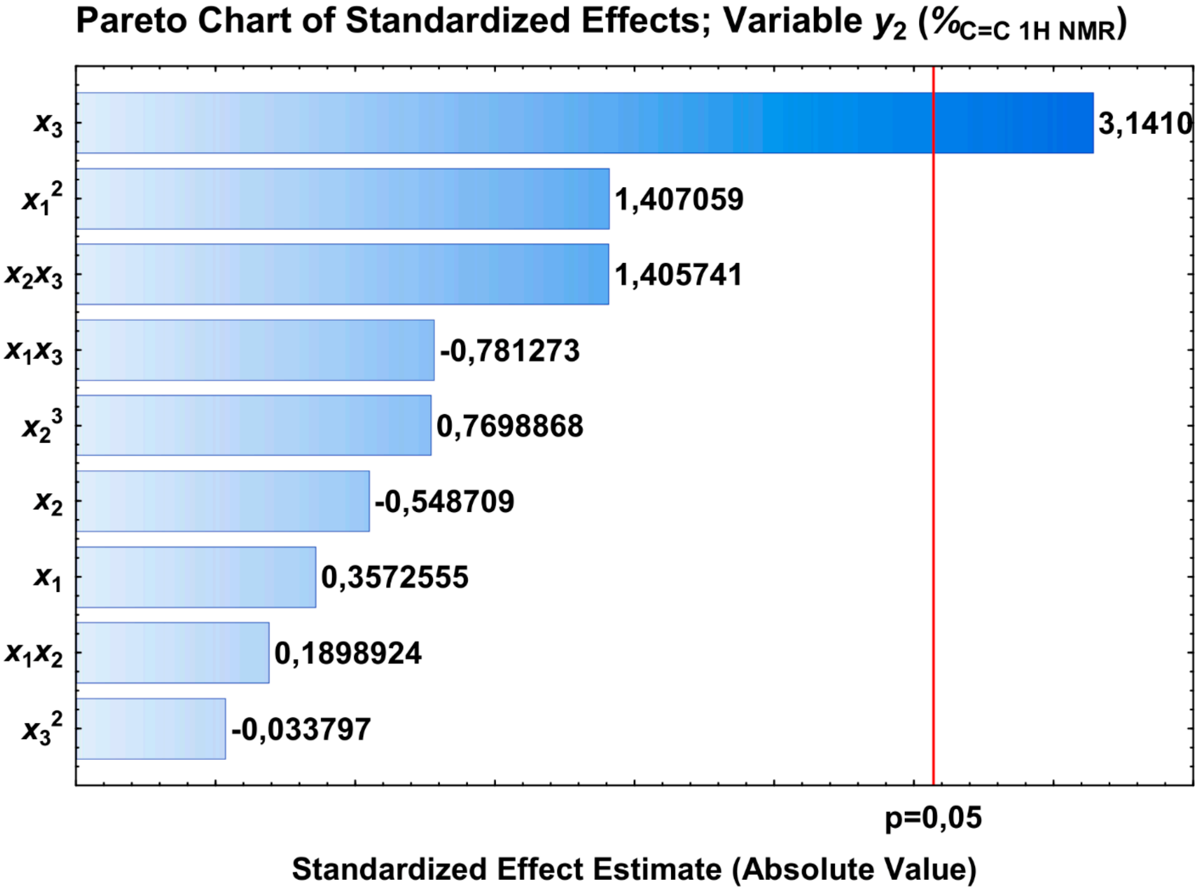

Figure S4. Pareto Chart of Standardized Effects for the $\%_{C=C\ 1H\ NMR}$ (y_2) variable.

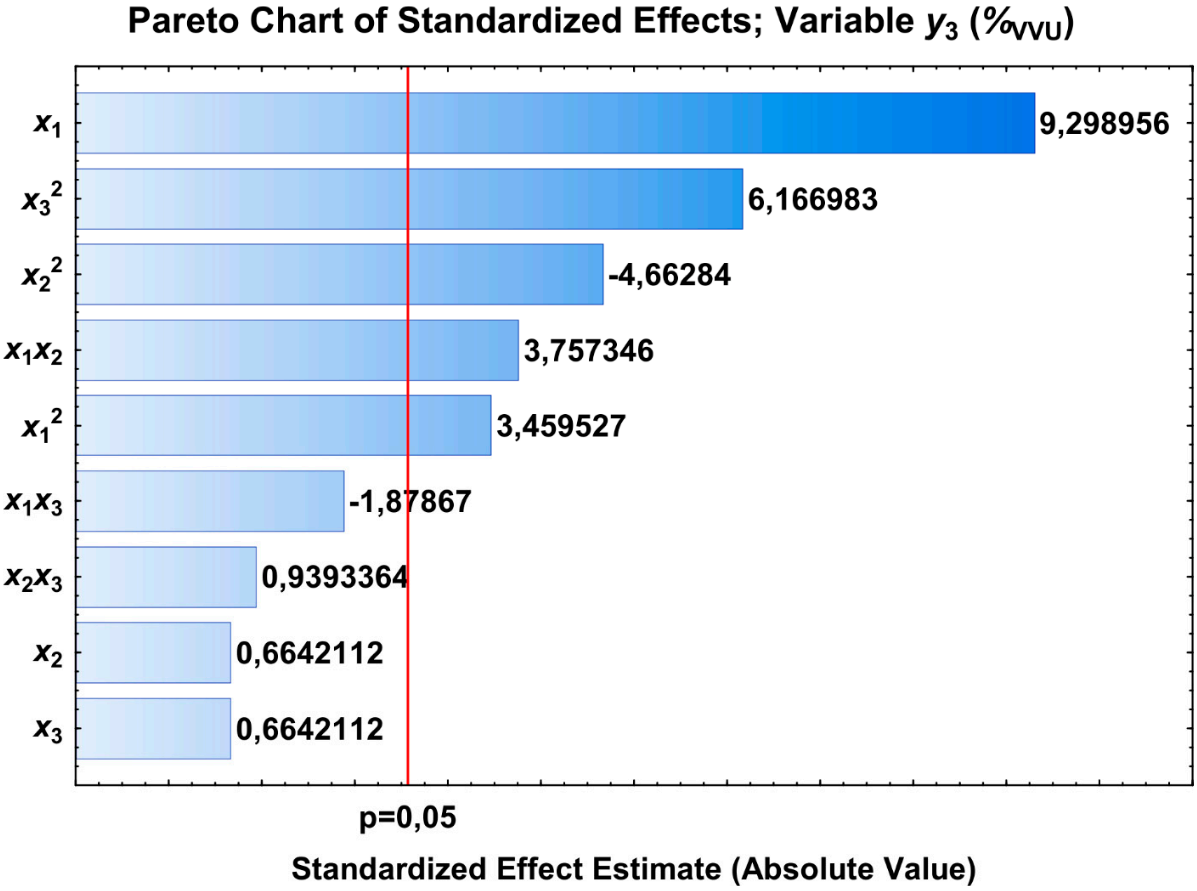

Figure S5. Pareto Chart of Standardized Effects for the $\%_{VVU}$ (y_3) variable.

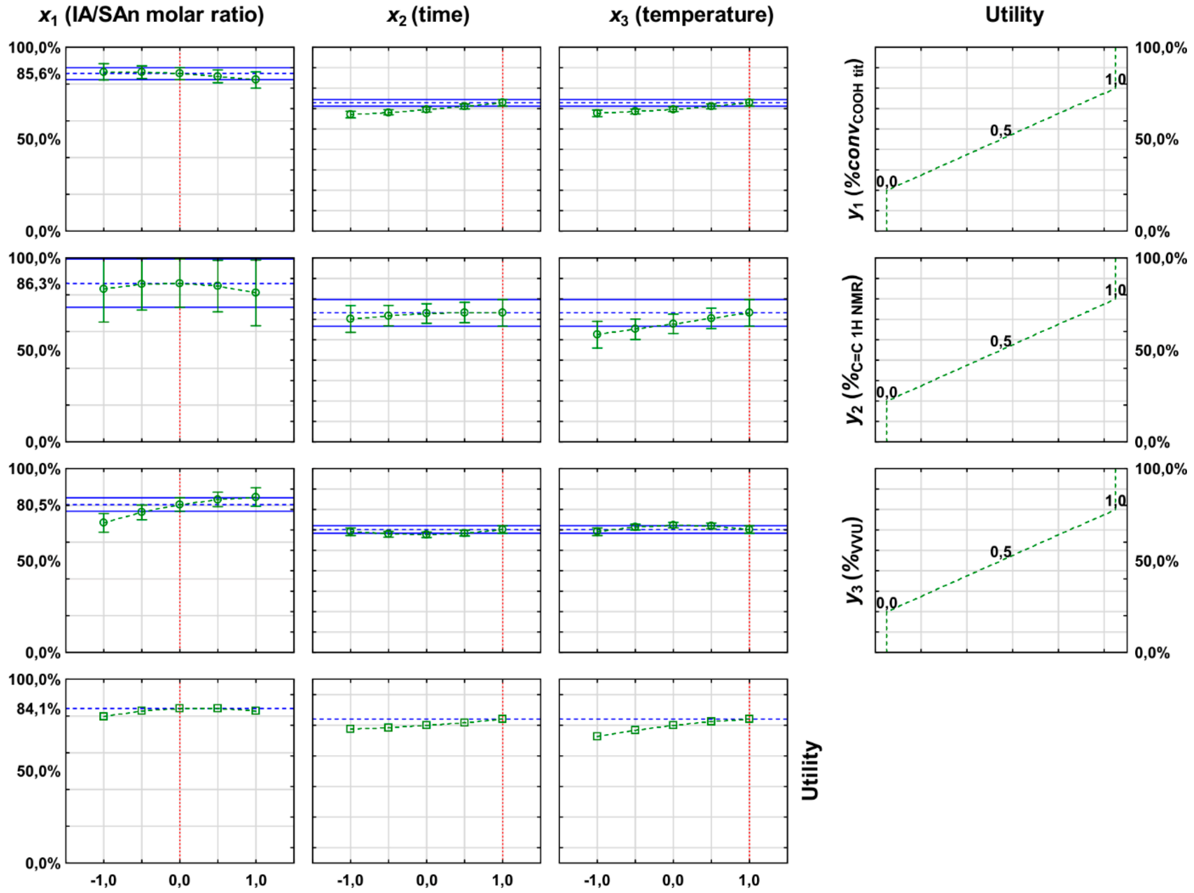

Figure S6. Profile of the approximated values of input variables and utility of the used mathematical model.

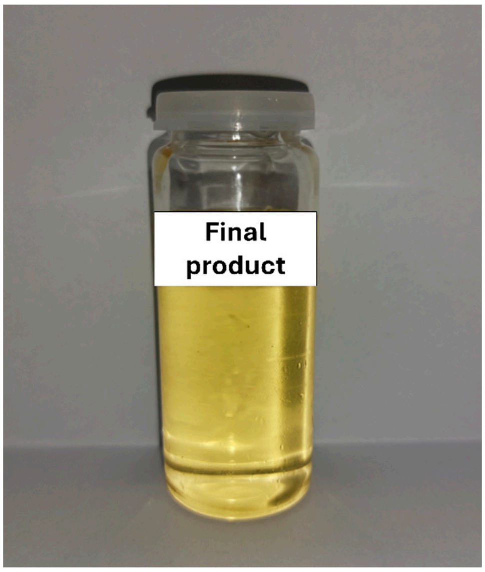

Figure S7. Consistency of the optimal product.

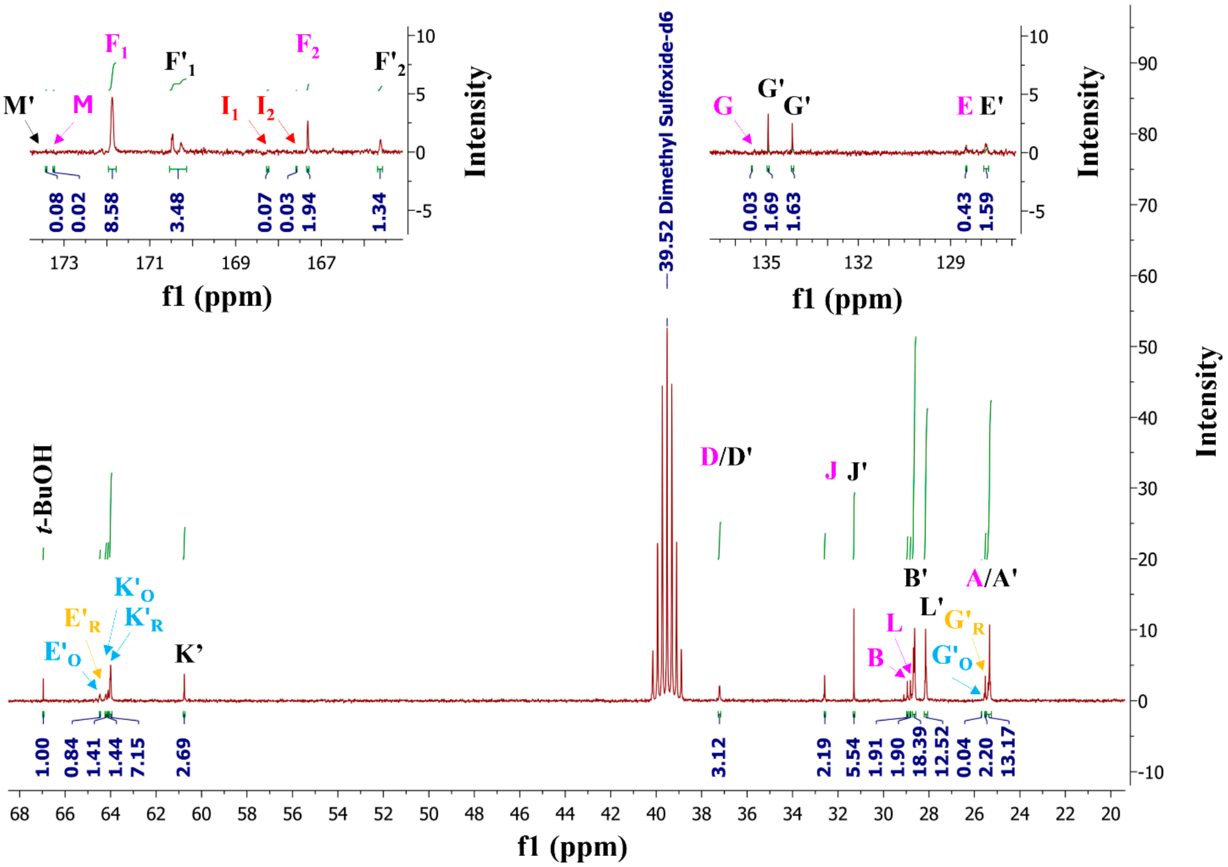

Figure S8. ^{13}C NMR spectra of poly(octamethylene itaconate-*co*-succinate).

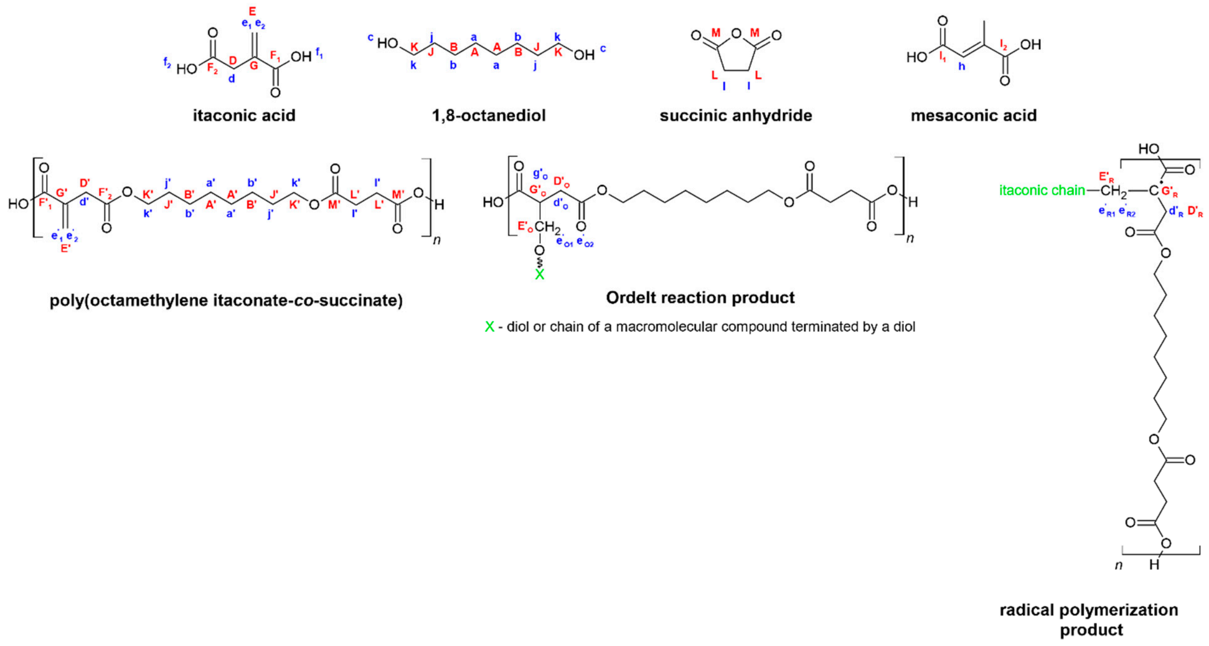

Figure S9. Assignment of protons and carbon atoms to the corresponding signals on the ^{1}H NMR and ^{13}C NMR spectra.

Table S1. Coded input variables in the Box-Behnken plan for the PISO synthesis optimization.

Input variable	Lower limit	Value of the coded input variable			Upper limit	Step of the input variable
		-1	0	1		
x_1	0	0.35:0.65	0.50:0.50	0.65:0.35	1	0.15
x_2	1	3	5	7	9	2
x_3	120	130	140	150	160	10

Table S2. Characterization of the polycondensation products.

No.	Coded Variable			GPC				^{1}H NMR Analysis					Titration Analysis		Viscosity [Pa·s]	
	x_1	x_2	x_3	M_n [g/mol]	M_w [g/mol]	*DI*	*RU*	$\%Ord_{NMR}$ [%]	$\%RP$ [%]	$\%Iz_{Mes}$ [%]	ED_{NMR} [%]	M_n [g/mol]	ED_{tit} [%]	$\%_{C=C\ IN\ tit}$ [%]	T = 25°C	T = 36.6°C
**1**	-1	-1	0	586	1244	2.1	6	20.8	8.2	3.3	61.5	627.8	66.5	88.8	65.6	18.8
**2**	1	-1	0	504	1035	2.1	5	19.9	6.5	2.3	64.6	570.8	71.9	70.6	286.2	38.8
**3**	-1	1	0	838	3370	4.0	16	21.5	8.5	3.4	61.6	918.3	76.8	80.9	160.1	24.9
**4**	1	1	0	569	1198	2.1	6	17.9	7.1	2.6	68.8	601.6	77.1	58.1	36.5	10.3
**5**	-1	0	-1	609	1941	3.2	9	20.7	8.7	2.6	60.1	677.2	72.0	99.9	8.1	2.2
**6**	1	0	-1	514	1097	2.1	5	19.0	6.8	2.3	65.4	568.9	67.6	68.8	476.7	91.5
**7**	-1	0	1	930	2927	3.1	14	11.9	8.1	3.4	76.4	986.5	85.7	92.4	857.1	61.2
**8**	1	0	1	773	1682	2.2	8	17.9	7.5	2.1	68.5	832.0	74.6	65.3	28.0	5.6
**9**	0	-1	-1	481	821	1.7	4	19.5	7.9	2.5	48.4	545.5	69.0	81.3	12.4	0.6
**10**	0	1	-1	557	1142	2.0	5	34.4	5.9	2.3	53.9	601.3	73.4	74.2	61.6	4.7
**11**	0	-1	1	601	1558	2.6	7	10.0	4.3	3.0	78.1	673.0	64.1	73.7	14.4	1.9
**12**	0	1	1	1013	2849	2.8	13	8.4	2.4	0.5	87.5	1078.0	85.2	67.5	14.9	3.6
**13**	0	0	0	718	1464	2.0	7	13.3	8.8	2.4	72.3	732.4	72.5	83.2	48.5	4.0
**14**	0	0	0	626	2147	3.4	10	14.4	8.6	2.7	70.3	703.7	73.4	82.7	19.9	2.9
**15**	0	0	0	691	1741	2.5	8	15.2	2.5	3.7	74.6	711.3	73.4	83.9	12.8	3.6
**Optimal product**	**0**	**1**	**1**	**1001**	**3129**	**3.1**	**14**	**8.7**	**2.6**	**0.1**	**87.3**	**1067.6**	**85.6**	**68.7**	**14.4**	**3.6**

Where: M_n – number-average molecular weight; M_w – weight-average molecular weight; ***DI*** – dispersity index; ***RU*** – number of repeating units; $\%Ord_{NMR}$ – percentage contribution of the Ordelt reaction; ***%RP*** – percentage contribution of the radical polymerization reaction; $\%Iz_{Mes}$ – percentage contribution of the isomerization reaction; $ED_{NMR\ /\ tit}$ – esterification degree (by NMR/titration); $\%_{C=C\ IN\ tit}$ – percentage of unreacted C=C double bonds.

Table S3. Values of the output variables to generate the response utility profile.

Utility	Output variable		
	conv$_{COOH\ tit}$ [%]	%$_{C=C\ 1H\ NMR}$ [%]	%$_{VVU}$ [%]
**Low**	0.00	0.00	0.00
**Medium**	50.0	50.0	50.0
**High**	100	100	100

Table S4. Significance test of regression equation coefficients for the investigated output variables (numbers are written to four significant digits).

Output variable	Input variable/Parameter relation	Regression coefficient	Standard error	t(5)	p	-95.00% Confidence limit	+95.00% Confidence Limit
y_1	*Constant*	0.7340	0.0047	155.6	0.0000000002080	0.7219	0.7461
	x_1	-0.08560	0.01156	-8.308	0.0007055	-0.1153	-0.05590
	x_1^2	0.01514	0.008504	1.557	0.1351	-0.006717	0.03700
	x_2	0.1112	0.01156	10.80	0.0002051	0.08153	0.1409
	x_2^2	-0.006030	0.008504	-1.235	0.5100	-0.02789	0.01583
	x_3	0.09971	0.01156	9.677	0.0003452	0.07000	0.1294
	x_3^2	-0.007718	0.008504	-1.457	0.4057	-0.02958	0.01414
	x_1x_2	0.03954	0.01634	2.714	0.06015	-0.002467	0.08155
	x_1x_3	0.001843	0.01634	0.1265	0.9146	-0.04016	0.04385
	x_2x_3	0.003979	0.01634	0.2730	0.8173	-0.03803	0.04599
y_2	*Constant*	0.7905	0.03396	23.28	0.000002722	0.7032	0.8777
	x_1	0.007429	0.02079	0.3573	0.7355	-0.04602	0.06088
	x_1^2	-0.04307	0.03061	-1.407	0.2184	-0.1217	0.03561
	x_2	-0.01141	0.02079	-0.5487	0.6068	-0.06486	0.04204
	x_2^2	-0.02356	0.03061	-0.7699	0.4761	-0.1022	0.05511
	x_3	0.06531	0.02079	3.141	0.02564	0.01186	0.1188
	x_3^2	0.001034	0.03061	0.03380	0.9743	-0.07764	0.07971
	x_1x_2	0.005584	0.02941	0.1899	0.8567	-0.07001	0.08118
	x_1x_3	-0.02297	0.02941	-0.7813	0.4700	-0.09857	0.05262
	x_2x_3	0.04134	0.02941	1.406	0.2188	-0.03425	0.1169
y_3	*Constant*	0.8021	0.009604	83.52	0.000000004664	0.7774	0.8268
	x_1	0.05469	0.005881	9.299	0.0002420	0.03957	0.06981
	x_1^2	-0.02995	0.008657	-3.460	0.01805	-0.05220	-0.007695
	x_2	0.003906	0.005881	0.6642	0.5360	-0.01121	0.01902
	x_2^2	0.04036	0.008657	4.663	0.005518	0.01811	0.06262
	x_3	0.003906	0.005881	0.6642	0.5360	-0.01121	0.01902
	x_3^2	-0.05339	0.008657	-6.167	0.001632	-0.07564	-0.03113
	x_1x_2	0.03125	0.008317	3.757	0.01319	0.009870	0.05263
	x_1x_3	-0.01563	0.008317	-1.8787	0.1191	-0.03700	0.005755
	x_2x_3	0.007813	0.008317	0.9393	0.3907	-0.01357	0.02919

Table S5. Model adequacy test for the investigated output variables.

ANOVA – Analysis Of Variance							
**Output variable**	**Source of variation**	**Sum of squares**	**Number of degree of freedom**	**Mean square**	$F_{calculated}$	$F_{critical}$	**Conclusion**
y_1	**Regression**	550.30	9	61.14	39.67	19.38	**Relevant**
	**Linear components**	454.65	3	151.55	98.32	19.16	**Relevant**
	**Non-linear components**	95.66	6	15.94	10.34	19.33	**Irrelevant**
	**Error**	84.74	5	-	-	-	-
	**Relevance error**	81.65	3	27.22	17.66	19.16	**Adequate**
	**Random error**	3.08	2	1.54	-	-	-
	∑	635.04	14	-	-	-	-
y_2	**Regression**	531.79	9	59.09	7.65	19.38	**Irrelevant**
	**Linear components**	356.10	3	118.70	15.37	19.16	**Irrelevant**
	**Non-linear components**	175.68	6	29.28	3.79	19.33	**Irrelevant**
	**Error**	172.95	5	-	-	-	-
	**Relevance error**	157.50	3	52.50	6.80	19.16	**Adequate**
	**Random error**	15.45	2	7.72	-	-	-
	∑	704.74	14	-	-	-	-
y_3	**Regression**	503.09	9	55.90	17.17	19.38	**Irrelevant**
	**Linear components**	241.70	3	80.57	24.75	19.16	**Relevant**
	**Non-linear components**	261.39	6	43.57	13.38	19.33	**Irrelevant**
	**Error**	13.83	5	-	-	-	-
	**Relevance error**	7.32	3	2.44	0.75	19.16	**Adequate**
	**Random error**	6.51	2	3.26	-	-	-
	∑	516.93	14	-	-	-	-

Table S6. Thermal analysis of POItcSc polycondensation product.

**Analysis**	**Characteristic temperature [°C]**				
**DSC**	T_{gh1}	T_{mh1}	T_{cc1}	T_{gh2}	T_{mh2}
	-52.5	24.1 -31.0	-8.40	-50.5	18.8-31.9
**TG**	$T_{d5\%}$	$T_{d30\%}$	$T_{d50\%}$	$T_{d85\%}$	**Heat resistance**
	231.2	364.6	392.6	416.9	152.5

Where: T_{gh1} – glass transition temperature during first heating; T_{mh1} – melting temperature during first heating; T_{cc1} – cold crystallization during cooling; T_{gh2} – glass transition temperature during second heating; T_{mh2} – melting temperature during second heating; $T_{d5\%}$ - 5% decomposition temperature; $T_{d30\%}$ - 30% decomposition temperature; $T_{d50\%}$ - 50% decomposition temperature; $T_{d85\%}$ - 85% decomposition temperature.

Equations Section:

To calculate the esterification degree (ED_{NMR}), isomerization to mesaconic compound contribution ($\%Iz_{\text{Mes}}$), Ordelt saturation contribution ($\%Ord$), the radical polymerization contribution ($\%RP$), and number-molecular weight (M_n), using ^{1}H NMR spectra, the following formulas were used:

$$\textbf{(1)}\ ED_{\text{NMR}} = \frac{\int e_1' + \int e_2'}{\int h + \int e_1' + \int e_1 + \int e_2' + \int e_2 + \int e_{O1}' + \int e_{O2}' + \int g_O' + \frac{1}{2}\times\int g_R'} \times 100\%$$

$$\textbf{(2)}\ \%Iz_{Mes} = \frac{\int h}{\int h + \int e_1' + \int e_1 + \int e_2' + \int e_2 + \int e_{O1}' + \int e_{O2}' + \int g_O' + \frac{1}{2}\times\int g_R'} \times 100\%$$

$$\textbf{(3)}\ \%Ord = \frac{\int e_{O1}' + \int e_{O2}' + \int g_O'}{\int h + \int e_1' + \int e_1 + \int e_2' + \int e_2 + \int e_{O1}' + \int e_{O2}' + \int g_O' + \frac{1}{2}\times\int g_R'} \times 100\%$$

$$\textbf{(4)}\ \%RP = \frac{\int g_R'}{\int h + \int e_1' + \int e_1 + \int e_2' + \int e_2 + \int e_{O1}' + \int e_{O2}' + \int g_O' + \frac{1}{2}\times\int g_R'} \times 100\%$$

$$\textbf{(5)}\ n = \frac{\int e_1' + \int e_2' + \frac{1}{2}\times\int d' + \frac{1}{8}\times\int(a'+b') + \frac{1}{4}\times\int j' + \frac{1}{4}\times\int k' + \frac{1}{4}\times\int l'}{7\times\frac{1}{9}\times\int t\text{-}BuOH}$$

$$\textbf{(6)}\ \overline{M_n} = n \times M_{RU}$$

Where: $\boldsymbol{M}_{\mathbf{RU}}$ – weight of the repeating unit of POItcSc (IA molar fraction: 0.65 = 227.79 g/mol; 0.50 = 225.28 g/mol; 0.35 = 220.78 g/mol); $\boldsymbol{n}$ – number of repeating units of POItcSc.

To calculate the statistical heat resistance value, the following formula was used:

$$\textbf{(7)}\ T_s = 0.49 \times [T_{d5\%} + 0.6 \times (T_{d30\%} - T_{d5\%})]$$
